# Discovery of plasma proteins and metabolites associated with left ventricular cardiac dysfunction in pan-cancer patients

**DOI:** 10.1186/s40959-025-00309-6

**Published:** 2025-02-13

**Authors:** Jessica C. Lal, Michelle Z. Fang, Muzna Hussain, Abel Abraham, Reina Tonegawa-Kuji, Yuan Hou, Mina K. Chung, Patrick Collier, Feixiong Cheng

**Affiliations:** 1 Genomic Medicine Institute, Lerner Research Institute, Cleveland Clinic, Cleveland, OH USA; 2 Department of Molecular Medicine, Cleveland Clinic Lerner College of Medicine, Case Western Reserve University, Cleveland, OH USA; 3 Department of Bioinformatics and Computational Biology, The University of Texas MD Anderson Cancer Center, Houston, TX USA; 4 Department of Gastrointestinal Medical Oncology, The University of Texas MD Anderson Cancer Center, Houston, TX USA; 5 Department of Cardiovascular Medicine, Heart, Vascular & Thoracic Institute, Cleveland Clinic, Cleveland, OH USA; 6 Cardiovascular and Metabolic Sciences, Lerner Research Institute, Cleveland Clinic, Cleveland, OH USA; 7 Case Comprehensive Cancer Center, Case Western Reserve University School of Medicine, Cleveland, OH USA; 8 Cleveland Clinic Genome Center, Lerner Research Institute, Cleveland Clinic, Cleveland, OH USA; 9 Section of Cardiovascular Imaging, Robert and Suzanne Tomsich Department Of Cardiovascular Medicine, Sydell and Arnold Miller Family Heart and Vascular Institute, The Cleveland Clinic Foundation, Cleveland, OH USA

**Keywords:** Cardiotoxicity, Machine learning models, Multi-omics, Plasma proteomics, Plasma metabolomics

## Abstract

**Background:**

Cancer-therapy related cardiac dysfunction (CTRCD) remains a significant cause of morbidity and mortality in cancer survivors. In this study, we aimed to identify differential plasma proteins and metabolites associated with left ventricular dysfunction (LVD) in cancer patients.

**Methods:**

We analyzed data from 50 patients referred to the Cleveland Clinic Cardio-Oncology Center for echocardiograph assessment, integrating electronic health records, proteomic, and metabolomic profiles. LVD was defined as an ejection fraction ≤ 55% based on echocardiographic evaluation. Classification-based machine learning models were used to predict LVD using plasma metabolites and proteins as input features.

**Results:**

We identified 13 plasma proteins (*P* < 0.05) and 14 plasma metabolites (*P* < 0.05) associated with LVD. Key proteins included markers of inflammation (ST2, TNFRSF14, OPN, and AXL) and chemotaxis (RARRES2, MMP-2, MEPE, and OPN). Notably, sex-specific associations were observed, such as uridine (*P* = 0.003) in males. Furthermore, metabolomic features significantly associated with LVD included 1-Methyl-4-imidazoleacetic acid (*P* = 0.015), COL1A1 (*P* = 0.009), and MMP-2 (*P* = 0.016), and pointing to metabolic shifts and heightened inflammation in patients with LVD.

**Conclusion:**

Our findings suggest that circulating metabolites may non-invasively detect clinical and molecular differences in patients with LVD, providing insights into underlying disease pathways and potential therapeutic targets.

**Supplementary Information:**

The online version contains supplementary material available at 10.1186/s40959-025-00309-6.

## Background

Cancer therapy-related cardiac dysfunction (CTRCD) is a significant and unpredicted consequence of cancer therapies leading to premature morbidity and death among cancer survivors [1]. Treatments such as anthracyclines, trastuzumab, and radiation therapy have been linked to cardiac adverse events, including left ventricular dysfunction (LVD) and heart failure [2–6]. CTRCD is defined by a decrease in the left ventricular ejection fraction (LVEF) of > 10%, to a value less than the lower limit of normal, confirmed by repeat imaging [7, 8]. Assessing the risk of CTRCD is challenging due to the variability in frequency of imaging or biomarker sampling. Moreover, the onset of CTRCD is unpredictable, occurring anywhere from the first year to nearly a decade after treatment onset [9]. Despite the known risk, official guidelines for monitoring high-risk patients taking known cardiotoxic therapies are lacking in the U.S.

Biomarkers, like N-terminal pro-B-type natriuretic peptide (NT-proBNP), and troponins are recommended to monitor cardiac function during cancer therapy [10, 11]. However, these biomarkers are the same as those used in cardiovascular disease (CVD) patients without a history of cancer. Therefore, the field warrants CTRCD-specific biomarker discovery. For example, myeloperoxidase is associated with repeated exposure to anthracyclines and trastuzumab [12–14]. However, many of these studies have only investigated targeted biomarker panels that may only include a dozen proteins or metabolites. Including a broader range of markers is warranted to encompass the full picture of the crosstalk between cancer and the cardiovascular system. Ample basic science data underscores crosstalk between cancer, cardiovascular, and metabolic disease which suggests that we should include more comprehensive biological variables into risk-calculator models [15–17]. Researchers have already investigated the utility of multi-omic biomarker discovery for cardiovascular disease [18–20]. Therefore, we believe that multi-omic biomarkers discovery approaches can be extended to determine CTRCD risk and is a promising approach in Cardio-Oncology [21–25].

Machine Learning models that use multi-omics features can help identify robust biomarkers for CTRCD [26, 27]. In recent years, the diagnostic ability of machine learning models has been evaluated to predict coronary artery disease [28], death after myocardial infarction [29], hospitalization following heart failure with preserved ejection fraction [30], echocardiograph assessment [31, 32], and detection of CTRCD [26, 33]. In this study, we applied classification models to assess the diagnostic capability of significant metabolites and proteins to distinguish cancer survivors with and without LVD. Compared to routine screening, we speculate that our multi-omics screening approach can improve upon cardiotoxicity surveillance of cancer survivors.

## Methods

### Study participants

All consecutive patients with a history of cancer and exposure to cytotoxic chemotherapy and who had a clinically necessary echocardiogram assessment from 12/2019 to 02/2022 were invited to enroll in our study (IRBs #11–194 and #19–1122) as described previously [34, 35]. Verbal and written informed consent were obtained. Participants were scheduled for a blood draw on the same day as their scheduled echocardiograph assessment. Blood samples were collected by registered healthcare professionals in the Lerner Research Institute Clinical Research Unit using collecting tubes containing EDTA. Vials were inverted 8–10 × and centrifuged at 2000 g for 15 min at 4 C°. Plasma was aliquoted in 500 ul increments and frozen at −80 C°.

### Clinical outcomes data acquisition

For each patient, 37 clinical variables commonly collected during cardio-oncology clinical practices were used in this study (Table 1, Supplemental Tables 1–3): (a) 10 general demographics; (b) 12 lab testing variables; (c) 8 cardiac variables; and (d) 7 cancer variables. Detailed clinical characteristics of the entire cohort used are provided in Table 1.

**Table 1:** Baseline cohort demographics

		*All*	*No LVD*	*LVD*
*n*		52	40	12
*Age (IQR)*		60.8 (50.0–71.0)	60.2 (49.1–70.0)	67.4 (54.2–71.2)
*BMI, kg/m*^*2*^*(IQR)*		27.4 (23.9–30.9)	27.0 (23.2–32.0	27.7 (24.8–29.7)
*Gender, n (%)*				
	Male	23 (44)	14 (38)	5 (36)
	Female	29 (56)	25 (63)	7 (64)
*Race, n (%)*				
	White	38 (73)	31 (78)	8 (67)
	African American	6 (12)	6 (15)	0 (0)
	Other	6 (12)	2 (5)	4 (33)
*Ejection Fraction (mean* ± *SD)*		60.0 ± 0.09	63.4 ± 0.04	47.90 ± 9.60
*Cancer stage, n (%)*				
	I	10 (19)	7 (18)	3 (25)
	II	9 (17)	7 (18)	2 (17)
	III	12 (23)	10 (25)	2 (17)
	IV	10 (19)	7 (18)	3 (25)
	Unknown	11 (21)	9 (23)	2 (17)
*CVD risk factors*				
	Smoker^*^	18 (35)	13 (33)	5 (42)
	Hyperlipidemia	19 (37)	14 (35)	5 (42)
	Diabetes	13 (25)	8 (20)	5 (42)
*Cardiotoxic drugs total mg * $(IQR)$* (n)*				
	Radiation	All locations Total Gy	952.2 (600.8–1355.6) (7)	1000 (825.4–1062.5) (3)
		Left chest Total Gy	95,037.5 (60,075–132780) (4)	0 (0)
	Anthracycline		363.5 (240.0–433.2) (9)	560 (511.6–572.5) (3)
	Trastuzumab	Total mg (n)	7470.6 (5300–54168) (12)	3975 (2362.5–5587.5) (2)
		Duration (months)	34 (11.0–44.0)	11.5 (11.25–11.5)
	Immunotherapy	Total mg	1400.0 (1)	448.8 (1)
		Duration (months)	13	13

*Abbreviations*: *IQR*, interquartile range, *LVD*, left ventricular dysfunction, *BMI*, body mass index, *SD*, standard deviation, *CVD* cardiovascular disease, *Gy* gray

*Smoker: current or past

LVD was used as the primary outcome and was defined by ejection fraction ≤ 55% by echocardiograph. LVEF was measured according to ASE guidelines using 2D or 3D echocardiography where appropriate, in an echocardiographic laboratory accredited by the Intersocietal Commission for the Accreditation of Echocardiography Laboratories (ICAEL), at a center of excellence. All studies were over-read by experienced board-certified echo-trained cardiologists with full access to all clinically relevant information within the electronic medical notes and the clinically reported ejection fraction was used in this study. We recognize that the ESC cardio-oncology guidelines and EACVI chamber quantification guidelines define left ventricular dysfunction (LVD) using different thresholds, often LVEF < 50%. Given the small number of cases meeting the criteria for overt ventricular failure (LVEF < 40%), we opted to focus on a broader definition of left ventricular dysfunction (LVD) rather than failure to ensure adequate representation and statistical feasibility. Cardiac outcomes defined by ICD 9/10 codes were manually checked by looking at patient charts on Epic for accuracy, including atrial fibrillation (AF), coronary artery disease (CAD), heart failure myocardial infarction (MI), and stroke. According to the diagnosis date of these 5 cardiac outcomes, we designated cardiac events diagnosed before cancer therapy as preexisting cardiac events, and those after cancer therapy as de novo LVD. All diagnoses defined by ICD 9/10 codes were further confirmed by a manual review of all medical records. All records to evaluate ejection fraction were taken after the start of therapy. A minority of participants had a record of an echocardiograph assessment within 6 months (6/51), or within two years (3/51) of their blood draw.

### Proteomics

Fifty participant samples were sent out for proteomics analysis (control = 40, LVD = 10). The selected frozen plasma samples were aliquoted in 100-μl samples and transferred to 96-well plates on dry ice, then sent to Olink proteomics for targeted proteomics analysis using the proximity extension assay technology. Olink multiplexing is based on the Proximity Extension Assay technology as previously described [36, 37]. Levels of 92 proteins were measured using the Olink Cardiovascular III panel, and the resulting data are provided in Normalized Protein eXpression (NPX) values. By internal cross-validation and interpolating controls, data are normalized and subjected to rigorous quality control.

### Metabolomics

Metabolome measurements were carried out by Human Metabolome Technologies, Inc., using the Q353 basic scan panel (control = 40, LVD = 10). Metabolite extraction from plasma samples, metabolome analysis using a capillary-electrophoresis time-of-flight mass spectrometer (CE-TOFMS), and data processing were all performed as described previously [38]. Briefly, sample processing and the analysis were conducted in randomized order. The signals in each sample were aligned under the tolerance of 100 p.p.m. in *m*/*z* and 0.5 min in migration time (MT). Detected peaks were annotated by comparison with a metabolome annotation library table, created based on the results of CE-TOFMS analysis of approximately 900 commercially available chemical standards. Peak areas were then evaluated and normalized to the area of internal standards (L-methionine sulfone and D-camphor-10-sulfonic acid in cation and anion modes, respectively; Solution ID, H3304-1002; HMT).

### Evaluation of machine learning models

In this study, we evaluated machine learning algorithms including logistic regression (LR), random forest (RF), gradient boosting (GB), support vector machine (SVM), decision tree (DT), and k-nearest neighbor (KNN) (Supplemental Table 6). The details of these approaches are provided in our previous study[26]. The models were trained, validated, and evaluated using the leave-one-out cross-validation (LOOCV) method. LOOCV is a special case of k-fold cross-validation, where k is equal to the number of samples in the dataset. Two patients were excluded from the 50 total patients due to not having both metabolomic and proteomic data available, leaving 48 patients for this analysis. A grid search was done to determine the optimal hyperparameters (Supplemental Table 7). Feature weights were determined using LR algorithm. Model performance was assessed using the area under the receiver operator characteristic (AUROC) and F1 score. To determine sensitivity and specificity, the threshold value producing the highest true positive rate and lowest false positive rate was chosen from the AUROC curve and the values were calculated accordingly (Supplemental Table 6). All machine-learning analyses were done using the Scikit-learn library version 1.0.2 in Python 3 [39].

### Statistical analysis

Student’s t-test or analysis of variance (ANOVA) was used for continuous normally distributed variables. The P-value for trend was computed with the use of Pearson correlation coefficient tests for continuous normally distributed, nonnormally distributed, and categoric variables, respectively. All analyses were performed with the use of R version 3.6.3 (R Foundation for Statistical Computing). Two-sided tests were used, and *P* < 0.05 was considered significant.

## Results

### Patient demographics

**Fig. 1:**
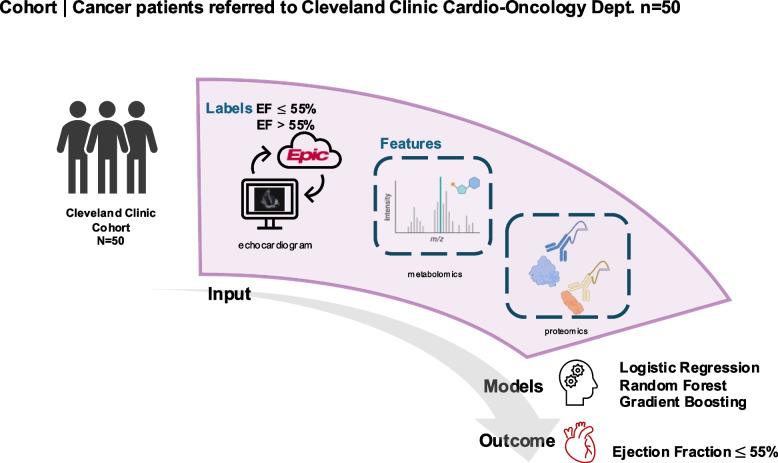
Study design overview. Patients with a history of cancer who were consulted to the Cardio-Oncology Center at Cleveland Clinic for echocardiograph assessment were asked to participate (*n* = 50). Plasma metabolites and proteins were used as features for the prediction of left ventricular dysfunction. We evaluated 6 classification methods including logistic regression, random forest, gradient boosting, k-nearest neighbor, decision tree, and support vector machine

52 patients referred to the cardio-oncology service at the Cleveland Clinic who met inclusion criteria were included in the study (Fig. 1). Patient demographics are listed in Table 1. Briefly, this study contained more females (*n* = 29, 56%) than males (*n* = 23, 44%) with an average age of 60.8 (IQR: 50.0–71.0) years and a BMI of 27.4 (IQR: 23.9–30.9 kg/m^2^). Participants identified as White (73%), African American (AA, 12%), or other (12%). Numerous cancer subtypes were reported, with the majority being breast (40%) and leukemia (12%) (Supplemental Table 1). The following cardiovascular disease risk factors were also reported: smoking (35%), hyperlipidemia (37%), and diabetes (25%). The majority of cancer therapy classes reported were chemotherapy (65%) and targeted therapy (40%) (Table 1).

Our cohort was comprised of 12 LVD cases and 40 controls. LVD was used as the primary outcome and was defined by ejection fraction ≤ 55% by echocardiograph (Methods). At the time of the study enrollment, 31 patients had pre-existing cardiac conditions and 21 patients developed CVD after their cancer diagnosis (Supplemental Table 2). The following CVD comorbidities defined by ICD9/10 codes and manually checked were reported: arrhythmia (control = 40%, LVD = 67%), coronary artery disease (control = 10%, LVD = 25%), heart failure (control = 3%, LVD = 50%), hypertension (control = 58%, LVD = 50%), myocardial infarction (control = 3%, LVD = 25%), and stroke (control = 5%, LVD = 8%). Data on routine labs were collected and shown in Supplemental Table 3. The only significant difference in labs was observed in NT-proBNP levels (P = 0.044) among LVD and control groups.

### Plasma protein dysregulation in left ventricular dysfunction cases

**Fig. 2:**
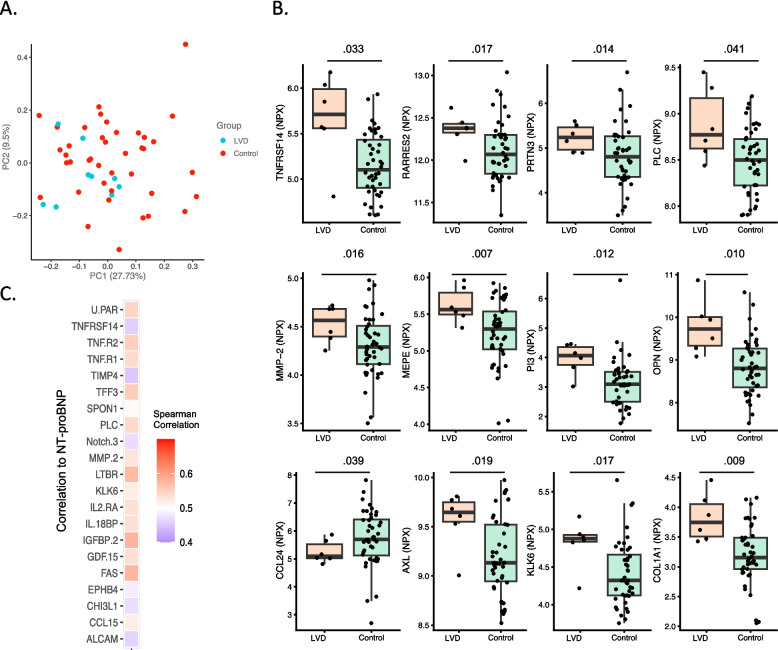
Plasma protein dysregulation in left ventricular dysfunction. **A** Principal component analysis of proteomics analysis for cancer survivors with cancer therapy-related cardiac dysfunction (*n* = 10, blue) versus without (*n* = 40, red). **B** Differentially expressed proteins (*P* < 0.05) from affinity proteomics analysis in patients with LVD versus control. **C** Heatmap correlating cardiovascular proteins associated with NT-proBNP (*P* < 0.05)

We observed circulating proteins clustered with LVD cases (Supplemental Fig. 1). Overall, principal component analysis showed LVD samples clustered together, and the control samples had more heterogenous expression (Fig. 2A). We identified 13 proteins significantly associated with EF ≤ 55%, including markers related to inflammation (ST2, TNFRSF14, OPN, and AXL) and chemotaxis (RARRES2, MMP-2, MEPE, and OPN) (Fig. 2B, Supplemental Fig. 1A, 2, Supplemental Information 2). Furthermore, we determined which proteins were most correlated with LVD by calculating the Pearson Correlation Coefficient (PCC) of each protein correlated with higher (top-quartile) or normal NT-proBNP expression (based on the panel NT-proBNP protein expression) and identified 21 significant (*P* < 0.05) proteins (Fig. 2C, Supplemental Fig. 3). We observed insulin growth factor binding protein 2 (IGFBP-2, P = 4.04 × 10^–6^), tumor necrosis family members (TNF-R1, *P* = 0.0001, TNF-R2, *P* = 0.001, FAS, *P* = 2.91 × 10^–8^, and LTBR, *P* = 0.0004, Fig. 2C, Supplemental Fig. 3) were highly associated. Furthermore, several proteins related to mechanosensing by endothelial cells or platelet activation were also associated with LVD (MMP2, *P* = 0.16, and COL1A1, *P* = 0.009, Fig. 2B). After multiple testing corrections, we did not observe any differential proteins, likely as a result of an unbalanced cohort. Nevertheless, the data suggest that LVD samples experience could experience extracellular matrix remodeling to enable increased inflammatory cell infiltration.

**Fig. 3:**
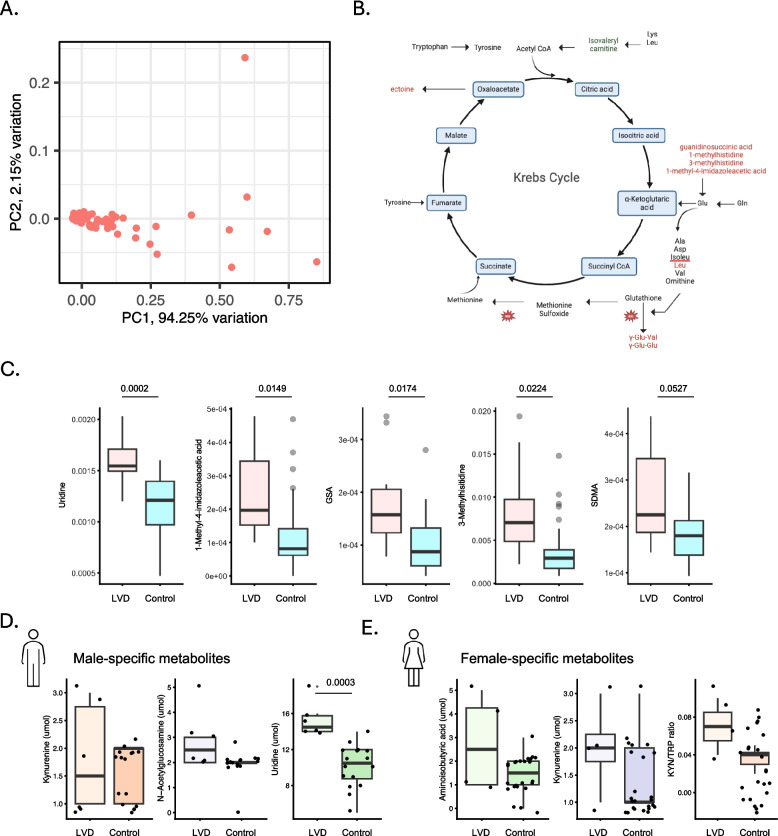
Plasma metabolite dysregulation in left ventricular dysfunction. **A** Principal component analysis of all significant metabolites combined. **B** Schematic of differentially expressed metabolites (red) converging to the glutamate synthesis pathway. **C** Overall top differentially expressed metabolites in LVD (*n* = 10) versus control patients (*n* = 40). **D**, **E** Sex-specific differentially expressed metabolites in males (control = 16, LVD = 6, **D**) and females (control = 22, LVD = 4, **E**)

### Discovery of targeted plasma metabolomic biomarkers for LVD

Furthermore, we next investigated whether similar or new biochemical patterns of LVD could be detected. We performed a targeted metabolomics panel of 353 polar metabolites. We identified 267 detectable metabolites, and 14 metabolites significantly (P < 0.05) differed in plasma from LVD patients and explained 94% of the variance among the samples (Fig. 3A, Supplemental Fig. 1B, Supplemental Information 3). Among these, we found a significant increase in metabolites relevant to muscle breakdown or cardiac remodeling including uridine and 3-methylhistidine (Fig. 3B-C). We also found metabolites relevant to inflammation and cancer progression like 1-methyl-4-imidazoleacetic acid, guanidinosuccinic acid (GSA), and symmetric dimethylarginine (SDMA) (Fig. 3B, C).

Recent work has indicated differences in cardiac biomarkers among males and females [40–42]. Therefore, we performed a sex-specific analysis and found 59 differentially expressed metabolites in males with LVD (Supplemental Fig. 4A, Supplemental Information 4) and 50 metabolites in females with LVD (Supplemental Fig. 4B, Supplemental Information 5). Among males, we found a significantly increased level of uridine (*P* = 0.003), and a trend towards increased N-acetylglucosamine, and kynurenine (Kyn) in male cases (Fig. 3D, SupplementalFig. 4A). Among female cases, we found a trend towards an increase in aminoisobutyric acid (*P* = 0.2505), kynurenine (*P* = 0.3925), and kynurenine/tryptophan (Kyn/Trp) ratio (*P* = 0.1747) in the LVD group (Fig. 3E, Supplemental Fig. 4B). Overall, this data corresponds with the inflammatory signatures more prominent in females and corroborating our findings seen in the proteomics data to suggest differences in LVD phenotypes by sex.

**Fig. 4:**
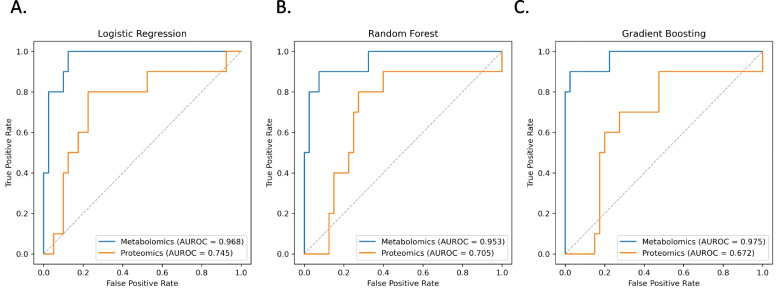
Evaluation of machine learning models to assess left ventricular dysfunction using plasma multi-omics biomarkers. **A**-**C** The area under the receiver operating curve (AUROC) for each classification model is shown. Differentially expressed metabolites (*n* = 3) (blue) and proteins (*n* = 14) (orange) were used as feature sets. The dotted line represents the theoretical baseline performance of a random feature. **A** Logistic Regression, **B** Random Forest, and **C** Gradient Boosting all achieved moderate to high performance

### Evaluation of machine leaning models for risk assessment of LVD using plasma multi-omics profiles

To determine if our identified circulating markers can predict cardiac dysfunction (ejection fraction, EF ≤ 55%), we trained six classification models (logistic regression (LR), random forest (RF), gradient boosting (GB), support vector machine (SVM), decision tree (DT), and k-nearest neighbor (KNN)) using two feature sets (differentially expressed metabolomics (*n* = 14) and proteomics (*n* = 5), Fig. 4 and Supplemental Fig. 5). Performance characteristics for each model are summarized in Supplemental Table 5. LR, RF, and GB models performed the best based on AUROC values (Fig. 4, Supplemental Fig. 5). For proteomics the AUROC values for each model were as follows- 0.745 for LR, 0.705 for RF, and 0.725 for GB. We observed that using significantly differentially expressed metabolites as features performed better with AUROC values of 0.968 for LR, 0.953 for RF, and 0.975 for GB. The random forest model maintained optimal sensitivity (90%) and specificity (90%) for differentiating between LVD cases and control (Supplemental Table 5). Similar model performance was observed when using LASSO, Elastic Net, and XGB Boost for feature selection (Supplemental Fig. 5). By performing LOOCV, we ensured that each sample in the dataset had an opportunity to be evaluated as a test case. This approach provides a reliable estimate of the model's generalization performance and helps to mitigate any bias that may arise from using a single train-test split. Furthermore, we determined that the metabolite features were enriched for glutamate metabolism, as well as fatty-acid and pyrimidine metabolism pathways (Fig. 3B). Our findings suggest that patients with lowered ejection fraction experience a shift in metabolic demands that are shown by increased glutamate production, which can consequently explain increased ROS-induced inflammation. Here, we show that the application of metabolomics-based machine-learning models may provide diagnostic utility to complement current cardio-oncology practices.

## Discussion

Researchers recognize the need to improve patient risk stratification techniques to enable early, non-invasive disease detection in cardio-oncology. Professional societies have also acknowledged the necessity for advancing cardiovascular risk assessment for cancer patients [43–46]. To date, biomarkers for myocardial injury are creatine kinase (CK)-MB, troponins, and natriuretic peptides [25]. These markers indicate the release of cardiac enzymes that result in irreversible damage hours after onset [11]. Numerous clinical trials are currently assessing biomarkers in cardio-oncology, underscoring the growing interest in leveraging clinical data to improve risk-calculator models [24]. This study aims to build upon the foundation of identifying putative CTRCD-related biomarkers, such as proteins and metabolites, for incorporation into risk prediction models, representing the first application of multi-omics-based machine learning models in cardio-oncology patients. We posit that this approach if validated in a larger cohort, can identify biomarkers that hold promise for identifying high-risk cancer patient groups who may require more intensive follow-up, enhanced surveillance, or tailored therapeutic strategies to mitigate their risk. Plasma markers allow for frequent, repeatable testing without imposing logistical burdens. This facilitates real-time monitoring of cardiac health during cancer treatment, enabling early detection of changes. Furthermore, we expect these outcomes to uncover critical biological mechanisms underlying CTRCD and related conditions. This could pave the way for the development of novel therapeutic targets, offering tailored interventions for this vulnerable patient niche.

In this study, we observed both proteins and metabolites associated with LVD cases indicated a signature of chronic inflammation and shifts in metabolic demand, including elevated levels of SDMA, methylimidazole acetic acid, GSA, TNF family members, and high Kyn/Trp ratio. Both oxidative stress and inflammation, established markers of cardiotoxicity, were prominent features. Notable, SDMA, conventionally a biomarker of renal dysfunction, has also been suggested as an independent biomarker for cardiovascular diseases, but not in CTRCD cases [47–52]. Additionally, elevated plasma levels of methylimidazole acetic acid and guanidinosuccinic acid (GSA) were observed in LVD cases, suggesting putative early artifacts of subclinical disease by increasing vascular permeability. Many of the metabolites that provide the largest weights in our ML models (GSA, 1-methylhistidine, 3-methylhistidine, 1-methyl-4-imidazoleacetic acid, $\gamma$-Glu-Val, and $\gamma$-Glu-Glu) converge to the glutamate metabolism pathway (Fig. 3B). Glutamate provides carbons to the tricarboxylic acid cycle (TCA) through α-ketoglutarate, which in turn is metabolized to amino acids, including alanine, aspartate, isoleucine, leucine, and valine- several of which are recognized markers of cardiovascular disease [53–55]. Our findings are consistent with previous studies. Thonusin et al*.* also noted increased glutamine in HER2-negative breast cancer patients 2-weeks after doxorubicin treatment, as well as reduced isovalerylcarnitine and isobutyrlcarnitine, which were significantly correlated with changes in LVEF and Troponin I respectively [56]. Similarly, in a cohort of 33 breast cancer patients treated with anthracyclines, increased TCA cycle intermediates (fumarate and succinate) and reduced tryptophan were observed in those who developed reduced ejection fraction [57]. While these findings highlight significant metabolic alterations linked to CTRCD, further longitudinal studies are essential to elucidate the temporal relationships between these metabolites and the onset and progression of disease.

Furthermore, patients with higher NT-proBNP also had increased circulating plasma levels of LTBR, tumor necrosis factor (TNF) family members like lymphotoxic beta receptor (LTBR), TNF receptors 1 and 2 (TNFR-1 and 2), and the anti-apoptotic receptor, Fas. Increased expression of Fas has been shown to increase with heart failure severity [58, 59]. Despite similar cardiac risk factors, patients with LVD exhibited an elevated inflammation profile not detectable by lab white blood cell counts alone, suggesting that metabolomics and proteomics markers are more sensitive in detecting subclinical disease.

Additionally, we observed differences in immune signatures in LVD cases, with a trend in elevated products from the kynurenine pathway, particularly prominent in female LVD cases. Pro-inflammatory cytokines activate tryptophan metabolism and, in turn, elevate circulating Kyn levels [60]. Kyn promotes T-regulatory cell differentiation and further increases anti-inflammatory cytokine production often seen in aging, psychiatric, and chronic cardiometabolic diseases [60, 61]. Furthermore, we observed elevated uridine levels in males with LVD. Although obesity and insulin resistance have been linked to elevated uridine levels both in animals and humans [62, 63], an increase in plasma uridine is also suggested to follow mild cardiac ischemia and reperfusion, as well as blood flow and arrhythmia [64]. The role of uridine in circulation is undetermined, but some data suggest that uridine may regulate vasodilation and can be a marker of insulin resistance [64, 65]. Sex differences in cardiovascular disease risk and clinical presentation have been well documented, but the biological explanations remain underexplored [66–68]. An expanded study of the Framingham cohort sought to investigate the sex differences in circulating biomarkers in CVD and found that 86% of the biomarkers were significantly different (FDR < 0.05) [41, 69]. For example, women with heart failure (HF) present later and are more likely to have preserved left ventricular ejection fraction and a non-ischemic etiology of HF [70]. However, we observed an increased Kyn/Trp ratio in females which is typically a result of indoleamine 2,3-dioxygenase-1 (IDO1) activation, a byproduct of elevated pro-inflammatory cytokine signaling like interleukin-1B, lipopolysaccharides, and tumor necrosis factor (TNF) [61]. Collectively, our findings, along with recent findings, underscore the urgency for future cardio-oncology studies and clinical practices to consider sex-specific risk assessment.

Finally, we applied classification machine learning models to test the diagnostic capability of distinguishing LVD cases from controls using differentially expressed proteins and metabolites. All outcomes received relatively high AUROC, outperforming proteomic features alone, suggesting the potential of machine learning models for early detection of LVD specific to patients with a history of cancer. Machine learning models will become increasingly important as next-generation sequencing and big data science become more integral to healthcare research. Therefore, optimizing ‘smart’ methods to complement cancer management will be vital for the future of cardio-oncology practices. Historically, biomarker discovery studies in cardio-oncology were focused on small targeted cardiac protein panels [13, 14, 71]. Our study applied an unbiased biomarker discovery approach using a metabolite panel of 365 metabolites and 90 protein markers, vastly surpassing the markers studied in cardio-oncology cohorts. Expanding the scope of variables fed into machine learning models will lead to more novel discoveries for understanding mechanisms of cardiotoxicity and understanding the ‘full picture.’ However, we acknowledge that the lack of reproducibility across Cardio-Oncology cohorts can stem from variability in the methodologies used for biomarker detection and will require standardized approaches of metabolites and proteins. Earlier proteomic studies have used traditional blood-derived cardiovascular panels, whereas current studies are using next-generation platforms, including proximity extension assays. Additionally, several of the few studies that have sampled plasma metabolites of CTRCD patients or research models have all used different metabolite detection methods like HPLC/MS, LC/Q-TOF MS, or capillary-electrophoreses-TOF/MS. The technical differences can explain differences in assay sensitivity, particularly for low-mass metabolites that are difficult to detect. Additionally, more attention will have to be placed on defining the time of evaluation, feature weights, and sample population to support reproducibility [24, 72].

### Limitations

It is difficult to assess whether the biomarkers we have depicted can provide early detection of CTRCD. Prospective studies paired with functional and longitudinal data of patients before diagnosis of CTRCD will be necessary to assess causality and will reduce the risk of overfitting machine learning models, especially for studies that include less than 100 individuals. Our cohort is limited by being imbalanced between disease and control cases and includes several cancer subtypes and cancer drug exposures. We acknowledge a potential limitation posed by the small sample size (n = 10), which prevents the use of a separate test set, as well as the inclusion of several cancer subtypes and cancer drug exposures. To address this limitation, we used a leave-one-out cross-validation (LOOCV) approach. This method is well-suited for small datasets, as it maximizes the use of available data while providing robust model validation. Furthermore, we paired our findings with literature references that support that these markers have implications in cancer or cardiovascular disease. Future work will require independent validation and the inclusion of data augmentation techniques to expand on existing patient data. Caution will be required for careful data harmonization and correction for confounding variables. Furthermore, most of the studies that have reported plasma metabolites or proteins in CTRCD patients have different assay methodologies, as previously discussed. Nonetheless, these findings provide a new biological perspective to cancer survivors with LVD.

## Conclusion

Overall, we demonstrate that multi-omic profiling can reveal underlying inflammation and matrix remodeling markers that are not captured in traditional laboratory tests. We identified abnormalities in circulating proteins and metabolites that stratified cancer survivors by those with and without LVD. Applying advanced machine learning models can help validate whether matrix remodeling and inflammation-related metabolites and proteins can improve LVD detection.

## Supplementary Information

Supplementary Material 1.

Supplementary Material 2.

Supplementary Material 3.

Supplementary Material 4.

Supplementary Material 5.

## Data Availability

The plasma derived metabolomics and proteomics data reported here are available as supplemental files (see Supplementary Files 2–5). All original code used to generate our machine learning models are publicly available and can be accessed at https://github.com/ChengF-Lab/CO-ML. Any additional information required to reanalyze the data reported in this paper is available from the lead contact upon request.

## Abbreviations

AF: Atrial fibrillation
ANOVA: Analysis of variance
AUROC: Area under the receiver operator curve
CAD: Coronary artery disease
CK-MB: Creatine kinase-MB
CTRCD: Cancer therapy-related cardiac dysfunction
CVD: Cardiovascular disease
DT: Decision Tree
EF: Ejection fraction
FDR: False discovery rate
GB: Gradient boosting
GSA: Guanidinosuccinic acid
HF: Heart failure
IDO1: Indoleamine 2,3-dioxygenase-1
KNN: K-nearest neighbor
Kyn: Kynurenine
LOOCV: Leave-one-out cross-validation
LVD: Left ventricular dysfunction
LVEF: Left ventricular ejection fraction
LR: Logistic regression
LTBR: Lymphotoxic beta receptor
MI: Myocardial infarction
NPX: Normalized Protein eXpression
NT-proBNP: N-terminal pro-B-type natriuretic peptide
PCC: Pearson correlation coefficient
SDMA: Symmetric dimethylarginine
SVM: Support vector machine
TNF: Tumor necrosis factor
Trp: Tryptophan
