# Supplementary material for: Discovery of plasma proteins and metabolites associated with left ventricular cardiac dysfunction in pan-cancer patients
Source: Cardiooncology. 2025 Feb 13;11:17. doi: 10.1186/s40959-025-00309-6 (PMC11823021; doi:10.1186/s40959-025-00309-6)
Supplement: Supplementary file 1 — Supplementary Material 1. [file 40959_2025_309_MOESM1_ESM.pdf]

Supplementary Information

Title: Discovery of plasma proteins and metabolites associated with left ventricular cardiac dysfunction in pan-cancer patients

Jessica C. Lal et al. *Cardio-Oncology* 2025

Supplementary Information contains 7 Supplementary Tables and 6 Supplementary Figures

Cancer subtypes, n (%)		*All*	*No LVD*	*LVD*
	Breast	21 (40)	18 (45)	3 (25)
	Colorectal	3 (6)	3 (8)	0 (0)
	Esophageal	4 (8)	3 (8)	1 (8)
	Gastrointestinal	1 (2)	1 (3)	0 (0)
	Kidney	1 (2)	1 (3)	0 (0)
	Leukemia	6 (12)	5 (13)	1 (8)
	Lung	2 (4)	1 (3)	1 (8)
	Lymphoma	2 (4)	2 (5)	0 (0)
	Mesothelioma	1 (2)	1 (3)	0 (0)
	Myeloma	3 (6)	1 (3)	2 (17)
	Ovarian	2 (4)	2 (5)	0 (0)
	Prostate	1 (2)	0 (0)	1 (8)
	Sarcoma	3 (6)	1 (3)	2 (17)
	Skin	1 (2)	0 (0)	1 (8)
	Thymus	1 (2)	1 (3)	0 (0)

Supplemental Table 1. Study cohort cancer subytpes.

		All	*Control*	*LVD*
CVD comorbidities, n (%)				
	Arrhythmia	24 (46)	16 (40)	8 (67)
	CAD	7 (13)	4 (10)	3 (25)
	Heart Failure	7 (13)	1 (3)	6 (50)
	Hypertension	29 (56)	23 (58)	6 (50)
	MI	4 (8)	1 (3)	3 (25)
	Stroke	3 (6)	2 (5)	1 (8)

Abbreviations:CVD: cardiovascular disease; CAD: coronary artery disease; MI: myocardial infarction; LVD: left ventricular dysfunction

Supplemental Table 2. Study cohort cardiovascular comorbidities.

		No LVD	*LVD*	*P-value*
		N=39	N=12	
Labs	Calcium (mg/dL)	9.43 ± 0.61	9.25 ± 0.32	0.186
	Glucose (mg/dL)	102.87 ± 23.18	104.00 ± 35.74	0.905
	WBC (k/uL)	5.82 ± 2.43	5.82± 2.43	0.833
	Platelets (k/uL)	232.31± 81.90	208.42± 56.33	0.263
	Neutrophils (k/uL)	3.77± 1.99	4.028± 1.78	0.692
	Lymphocytes (k/uL)	1.23± 0.63	0.95± 0.44	0.080
	Monocytes (k/uL)	0.50± 0.22	0.54± 0.16	0.544
	Potassium (mmol/L)	6.74 ± 16.15	4.26 ± 0.40	0.346
Biomarkers (IQR)	Median Troponin T (ng/L)	0.01 (0.01-0.015)	0.23 (0.063-4.035)	0.288
	Median Troponin I (ng/mL)	0.02 (0.01-0.02)	9.51 (4.761-14.254)	0.501
	Median NT-proBNP (pg/mL)	93.00 (85.00-161.00)	946.00 (612.75-2091.5)	0.044
	Median hsCRP(mg/L)	4.00 (1.80-100.40)	7.50 (5.00-155.50)	0.735

Abbreviations:LVD, left ventricular dysfunction; IQR, inner quartile range; WBC, white blood cells; NT-proBNP, N-terminal-pro-B-type natriuretic peptide; hsCRP, high sensitivity c-reactive protein

Supplemental Table 3. Clinical laboratory markers in patients with/without cancer therapy related left ventricular dysfunction.

Metabolite	FC	P-value	q-value
Uridine	1.3808	0.0002	0.0440
Hexanoic acid	0.0000	0.0016	0.1598
γ-Glu-Val	1.3623	0.0018	0.1598
Indole-3-acetic acid	0.2207	0.0029	0.1918
Tyr	0.7990	0.0087	0.4652
1-Methyl-4-imidazoleacetic acid	2.0550	0.0148	0.5293
Isovalerylcarnitine	0.0000	0.0173	0.5293
Guanidinosuccinic acid	1.8426	0.0174	0.5293
Decanoic acid	2.1123	0.0200	0.5293
γ-Glu-Glu	1.5844	0.0200	0.5293
1-Methylhistidine<br>3-Methylhistidine	2.3086	0.0222	0.5293
Ethanolamine phosphate	1.4391	0.0238	0.5293
Ectoine	7.0702	0.0335	0.6477
Methionine sulfoxide	0.7488	0.0471	0.6477

Supplemental Table 4. Significant metabolites differentially expressed in LVD cases versus controls.

Protein	Fold change	P-value	q-value
ST2	0.9284	0.0158	0.5161
KLK6	1.0783	0.0262	0.5161
MMP-2	1.0439	0.0322	0.5161
TLT-2	1.0580	0.0453	0.5161
CHIT1	0.8562	0.0461	0.5161
COL1A1	1.1167	0.0527	0.5161

Supplemental Table 5. Significant proteins differentially expressed in LVD cases versus controls.

	Metabolomics			Proteomics		
	**LR**	**RF**	**GB**	**LR**	**RF**	**GB**
**Threshold**	0.252	0.280	7.83E-09	0.214	0.239	0.145
**Specificity**	0.900	0.900	0.875	0.775	0.750	0.725
**Sensitivity**	0.800	0.900	0.900	0.700	0.700	0.600
**F1**	0.727	0.783	0.750	0.538	0.519	0.444
**AUROC**	0.968	0.953	0.975	0.745	0.705	0.673

Supplemental Table 6. Performance characteristics for predicting LVD

Algorithm	Hyperparameter	Values
**Logistic Regression**	C	0.01, 0.1, 1, 10, 100, 1000
**Random Forest**	max_features	0.2, 0.4, 0.8, 1
	max_depth	4, 8, 12
	n_estimators	100, 500
**Gradient Boosting**	max_depth	2, 3, 4
	learning_rate	0.01, 0.05, 0.1
	subsample	0.75, 0.9, 1
**Support Vector Machine**	C	0.1, 1, 10, 100
	Gamma	$1x10^{-3}$, $1x10^{-2}$
**Decision Tree**	max_depth	2, 3, 4, 6
	min_samples_split	2, 4, 8
	min_samples_leaf	1, 2, 4
**K-nearest neighbors**	n_neighbors	3, 5, 7, 12
	Metric	"euclidean", "correlation"

Supplemental Table 7. Hyperparameters tested for each machine learning model

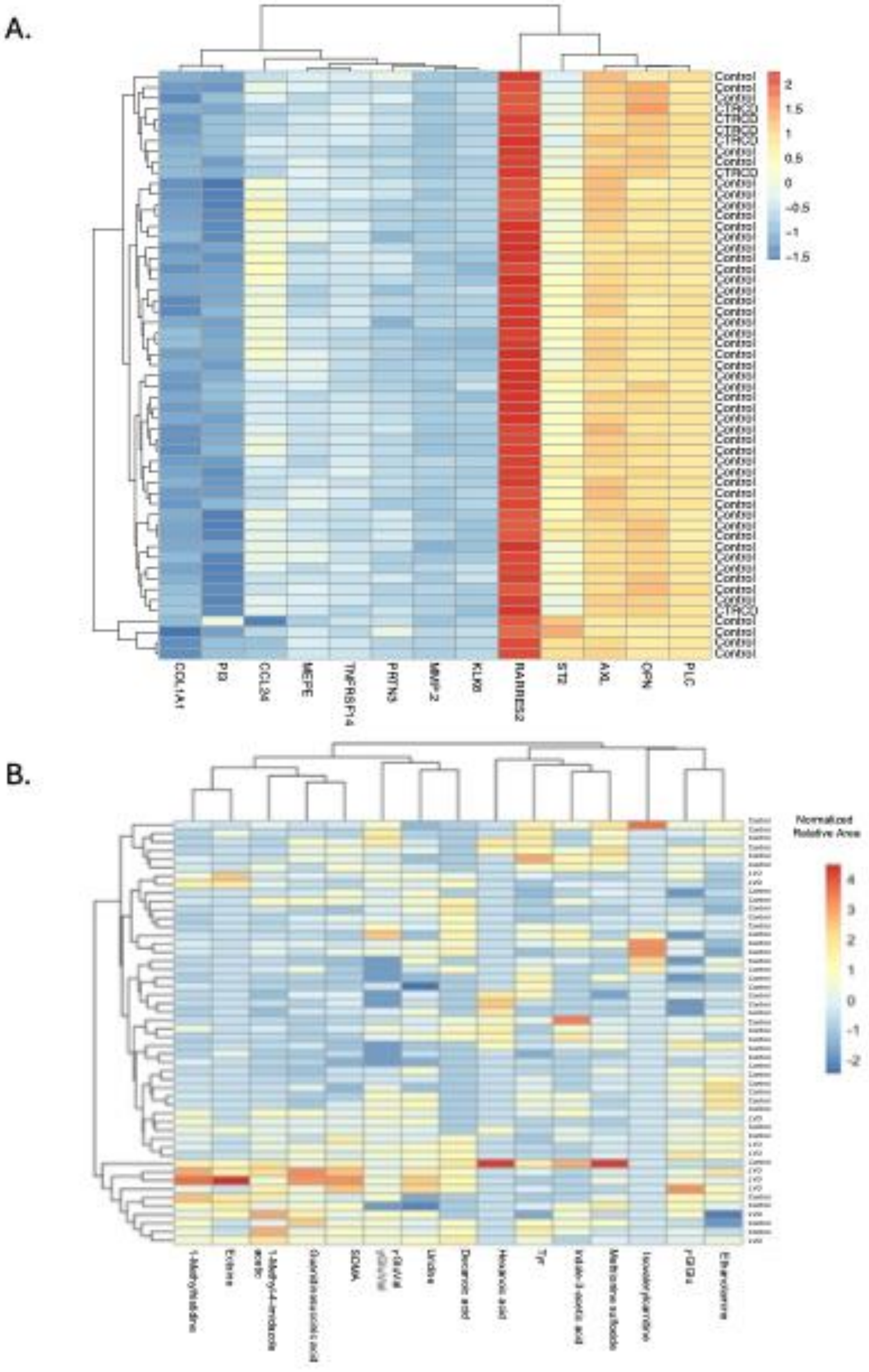

Supplemental Figure 1. Overview of differentially expressed proteins in LVD versus control. Heatmap and hierarchical clustering representing the top (A) proteins and (B) metabolites associated with left ventricular dysfunction (LVD).

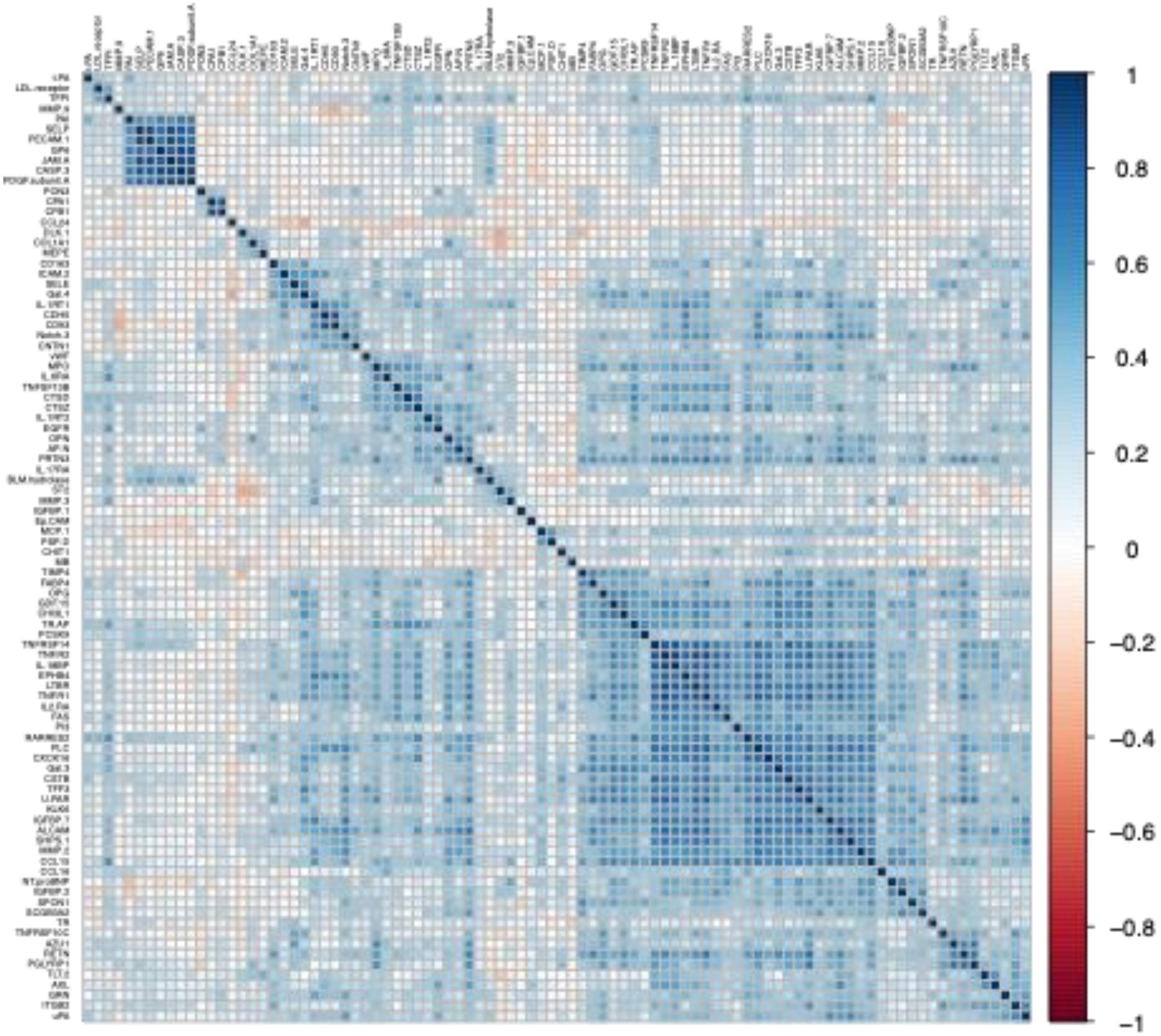

Supplemental Figure 2 Overview of differentially expressed proteins in CTRCD versus control. Pearson correlation coefficient matrix comparing co-correlation of detectable cardiovascular proteins in human plasma.

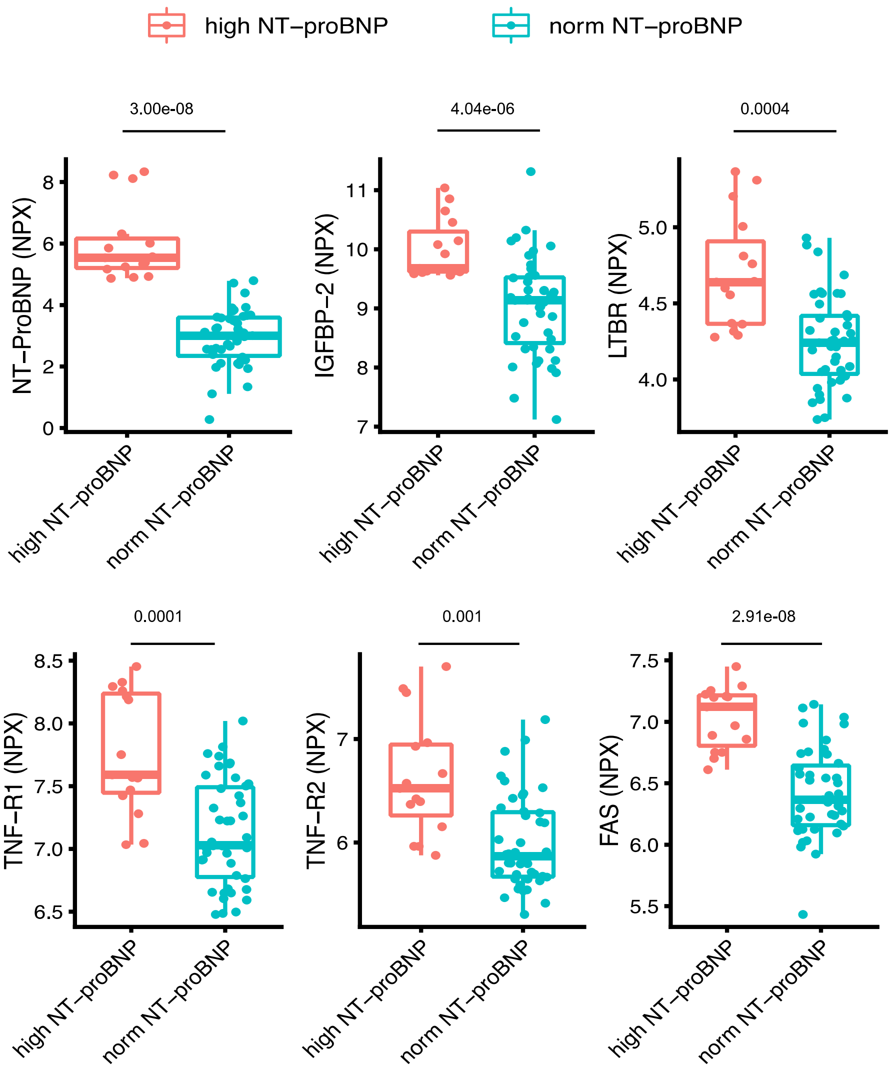

Supplemental Figure 3. Proteins correlated with higher NT-proBNP plasma levels. Samples with the highest quartile expression of NT-proBNP (red) according to affinity proteomics analysis were compared to samples with middle to lower quartile range in expression of NT-proBNP (blue). P-values are shown.

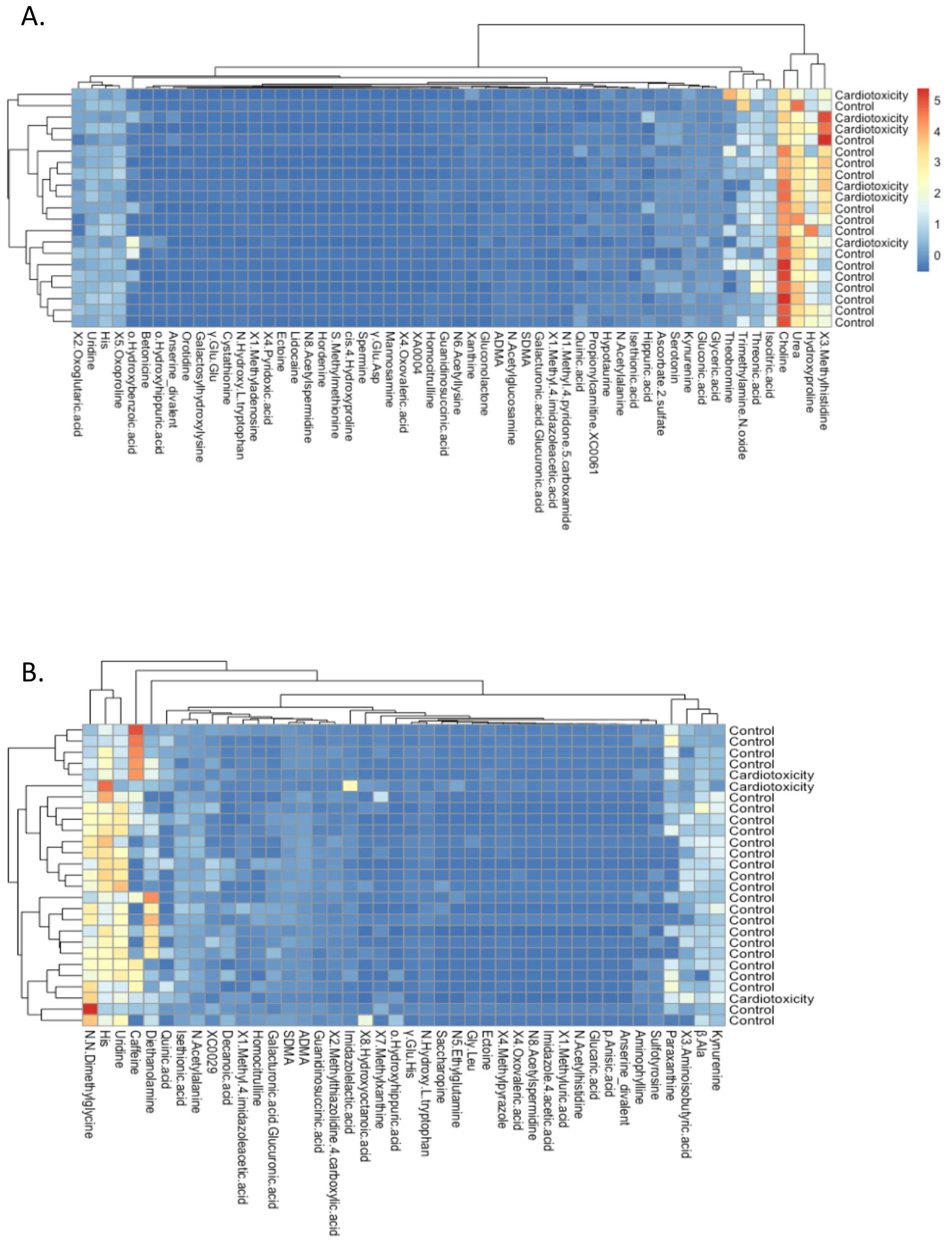

Supplemental Figure 4. Overview of sex-specific differentially expressed metabolites in LVD versus control. (A) Heatmap and hierarchical clustering representing the top proteins associated with LVD in (a) males and (b) females.

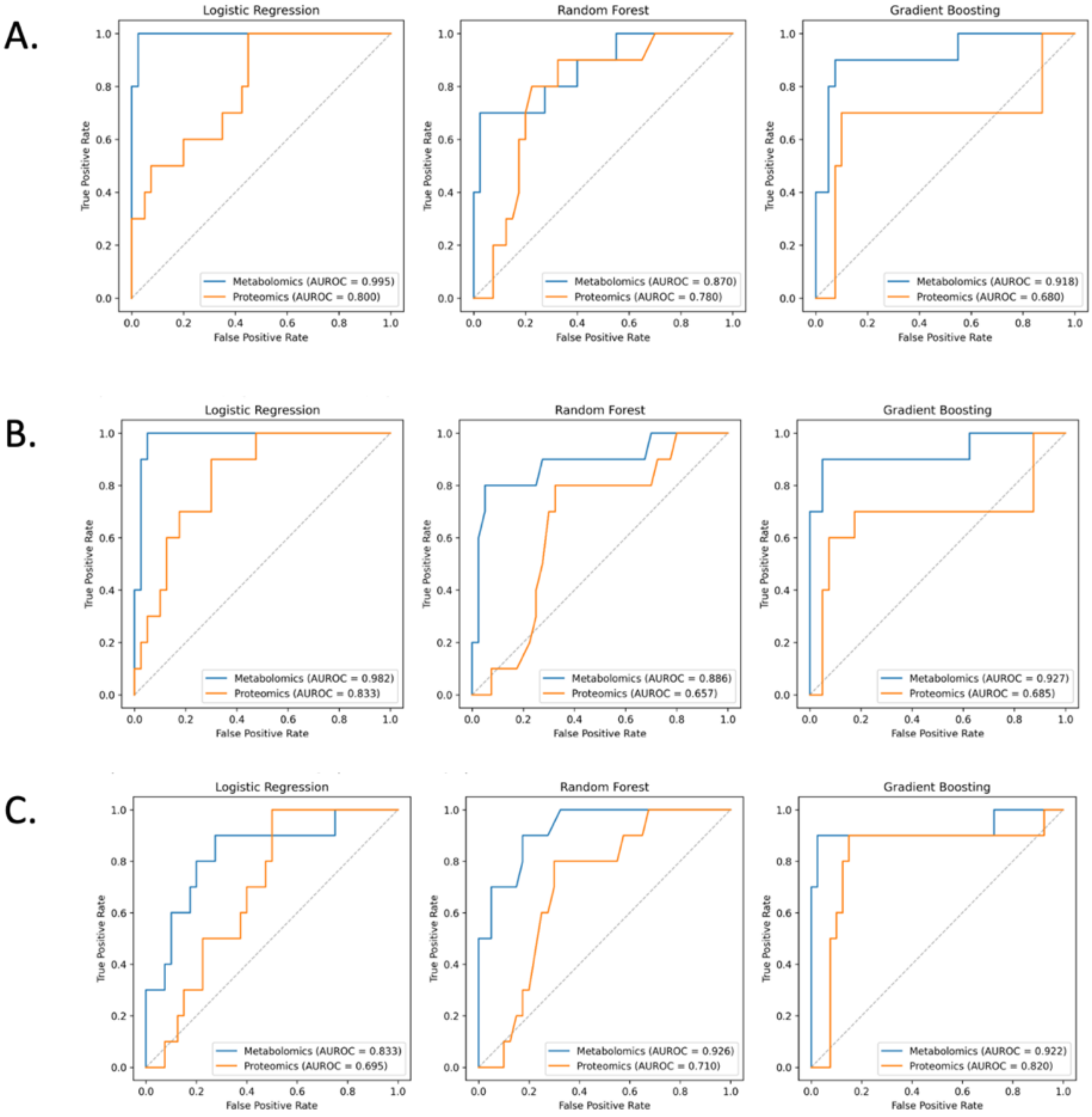

Supplemental Figure 5. Features selection for machine learning models to assess cancer therapy-related cardiac dysfunction using plasma multi-omics biomarkers. The area under the receiver operating curve (AUROC) Logistic Regression, Random Forest, and Gradient Boosting classification models are shown. Feature selection using (A) LASSO, (B) Elastic Net, and (C) XGB Boost were performed for model input. The dotted line represents the theoretical baseline performance of a random feature.

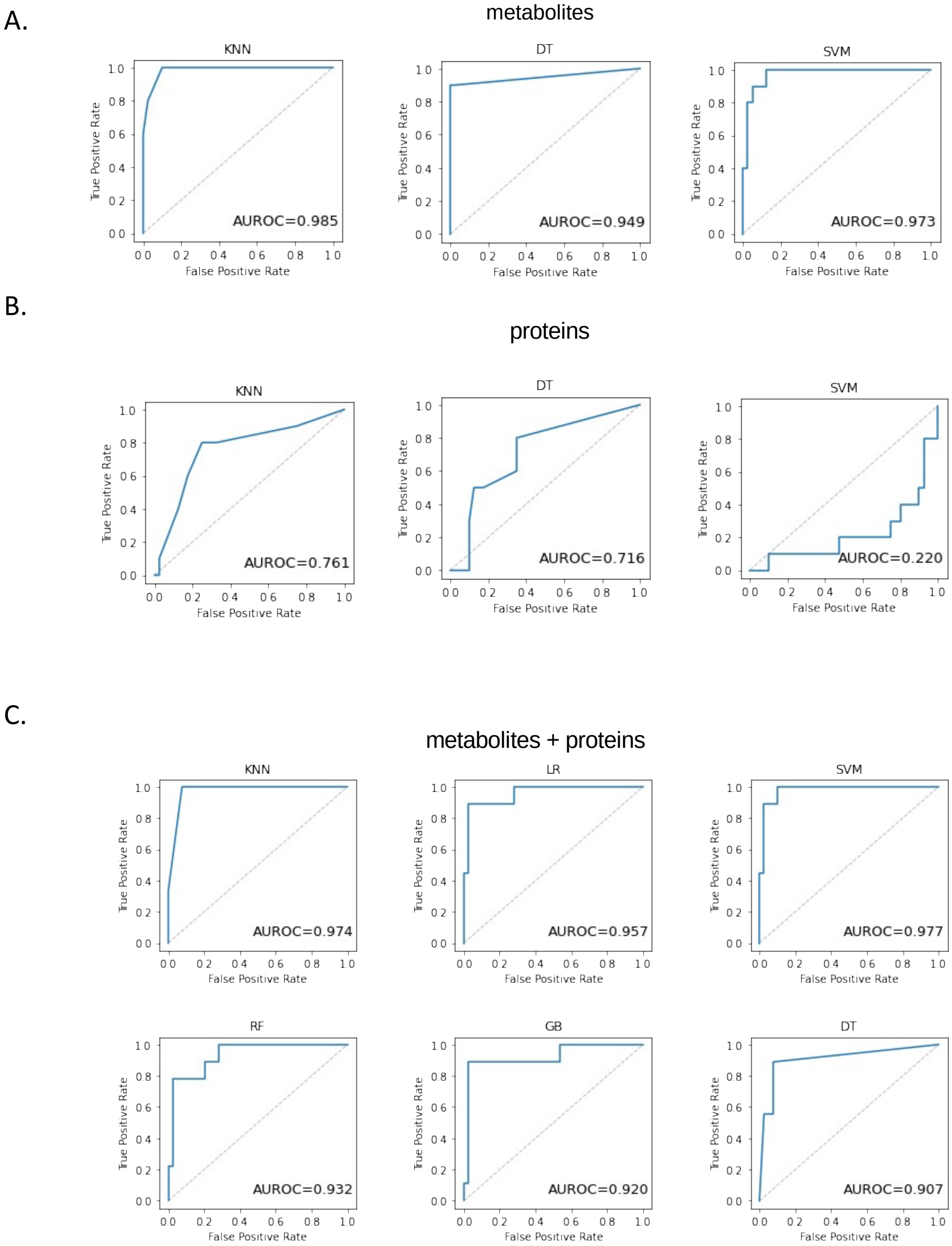

Supplemental Figure 6. Evaluation of machine learning models to assess cancer therapy-related left ventricular dysfunction using plasma multi-omics biomarkers. (A-B) The area under the receiver operating curve (AUROC) for k-nearest neighbor (KNN), decision tree (DT), and support vector machine (SVM) classification model is shown. Differentially expressed metabolites (A) and proteins (B) were used as feature sets. (C) Combined differentially expressed metabolites and proteins were tested for each classification model. The dotted line represents the theoretical baseline performance of a random feature.
